# Estimation of Health-Related Physical Fitness Using Multiple Linear Regression in Korean Adults: National Fitness Award 2015–2019

**DOI:** 10.3389/fphys.2021.668055

**Published:** 2021-05-13

**Authors:** Sung-Woo Kim, Hun-Young Park, Hoeryong Jung, Jinkue Lee, Kiwon Lim

**Affiliations:** ^1^Physical Activity and Performance Institute, Konkuk University, Seoul City, South Korea; ^2^Department of Sports Medicine and Science, Graduate School, Konkuk University, Seoul City, South Korea; ^3^Department of Mechanical Engineering, Konkuk University, Seoul City, South Korea; ^4^Department of Physical Education, Konkuk University, Seoul City, South Korea

**Keywords:** health-related physical fitness, multiple linear regression, cardiorespiratory fitness, hand grip strength, muscular endurance

## Abstract

Continuous health care and the measurement of health-related physical fitness (HRPF) is necessary for prevention against chronic diseases; however, HRPF measurements including laboratory methods may not be practical for large populations owing to constraints such as time, cost, and the requirement for qualified technicians. This study aimed to develop a multiple linear regression model to estimate the HRPF of Korean adults, using easy-to-measure dependent variables, such as gender, age, body mass index, and percent body fat. The National Fitness Award datasets of South Korea were used in this analysis. The participants were aged 19–64 years, including 319,643 male and 147,600 females. HRPF included hand grip strength (HGS), flexibility (sit and reach), muscular endurance (sit-ups), and cardiorespiratory fitness (estimated VO_2*max*_). An estimation multiple linear regression model was developed using the stepwise technique. The outlier data in the multiple regression model was identified and removed when the absolute value of the studentized residual was ≥2. In the regression model, the coefficient of determination for HGS (adjusted *R*^2^: 0.870, *P* < 0.001), muscular endurance (adjusted *R*^2^: 0.751, *P* < 0.001), and cardiorespiratory fitness (adjusted *R*^2^: 0.885, *P* < 0.001) were significantly high. However, the coefficient of determination for flexibility was low (adjusted *R*^2^: 0.298, *P* < 0.001). Our findings suggest that easy-to-measure dependent variables can predict HGS, muscular endurance, and cardiorespiratory fitness in adults. The prediction equation will allow coaches, athletes, healthcare professionals, researchers, and the general public to better estimate the expected HRPF.

## Introduction

Physical fitness is defined as a physiological state of wellbeing in which one can perform daily activities without strain, or that provides the basis for exercise performance. Health-related physical fitness (HRPF) includes components related to a health condition, such as musculoskeletal and cardiorespiratory fitness (CRF; Liguori and American College of Sports Medicine, 2020).

Health-related physical fitness and physical activity (PA) level are often used together, with physical fitness generally considered a more accurate measurement of PA level than self-reported assessments (Williams, 2001). PA involves body movements caused by skeletal muscle contractions that increase energy consumption beyond the basic level (Meredith and Welk, 2010; Liguori and American College of Sports Medicine, 2020). Systematic research on the association between PA and health conditions began six decades ago, and since then, the scientific literature has confirmed the relationship between these two areas (Liguori and American College of Sports Medicine, 2020). Physical fitness was reported to be similar to PA in terms of its association with morbidity and mortality (Blair and Brodney, 1999; Erikssen, 2001). However, physical fitness predicts health outcomes more strongly than PA (Blair et al., 2001; Williams, 2001; Myers et al., 2004). Previous studies have shown at least a 50% decrease in mortality among individuals with a high physical fitness level compared to those with a low physical fitness level (Myers et al., 2004). In addition to serving as a prognostic and diagnostic health indicator in clinical settings, CRF has been used as an indicator of regular exercise (Lin et al., 2015). Warburton et al. reported that the physiological functions of the human body and HRPF continuously decrease with aging, leading to an increased risk for chronic diseases (Warburton et al., 2006). Among the HRPF components, the CRF index’s maximal oxygen uptake decreases by about 3–6% due to aging (Fleg et al., 2005). High levels of HRPF maintained from adulthood can reduce musculoskeletal, cardiovascular, and metabolic diseases such as osteoporosis, sarcopenia, hypertension, and diabetes (Carnethon et al., 2003; Katzmarzyk et al., 2004; Barry et al., 2014; Kim et al., 2019b). The HRPF is an indirect health indicator of the body, and continuous care is important. Therefore, all of the previous study findings establish the need to include HRPF testing in health condition monitoring systems (Ortega et al., 2008). Furthermore, the World Health Organization suggested that regular physical fitness and PA testing should be examined as a public health priority (World Health Organization [WHO], 2010). To prevent chronic diseases, continuous healthcare is necessary, which requires the evaluation of HRPF. However, measurements of HRPF are often not practical or feasible to perform in daily life. Additionally, laboratory methods can accurately measure physical fitness, but may not be a feasible approach for entire populations owing to cost, time constraints, and the need for qualified technicians and sophisticated devices.

The American College of Sports Medicine suggested that physical health is a measurable result of an individual’s PA and exercise habits, which is why many healthcare providers value the accurate and precise measurement of HRPF (Liguori and American College of Sports Medicine, 2020). Common HRPF tests include the isometric hand grip strength (HGS) test for measuring muscle strength (Bäckman et al., 1995), the sit and reach test for flexibility (Mier, 2011), the sit-up test for abdominal muscular endurance (Chen et al., 2020b), and the graded exercise test for cardiorespiratory endurance (Beltz et al., 2016; Kim et al., 2019b). The association between HRPF and health conditions has been established in several studies (Mendes et al., 2016; Chrismas et al., 2019; Chen et al., 2020a). Recently, technological advances in health care and sports science have provided coaches, athletes, healthcare professionals, and researchers with efficient, reliable, and economical means to record health-related and exercise performance data (Seshadri et al., 2017; Aroganam et al., 2019; Kim et al., 2019a; Ray et al., 2019). The connected gains of novel analytical techniques, portable and reliable devices, and comprehensive software programs suggest that research on health promotion will increase in the future (Loncar-Turukalo et al., 2019). Several predictive equations have been developed to estimate HRPF to increase utility for field-based research (Esco et al., 2008; Shenoy et al., 2012; Lopes et al., 2018; Zaccagni et al., 2020). These previous studies generally linked HRPF parameters to laboratory evaluations. However, there were differences in the equation’s estimation reliability due to sample size, the number of independent variables, differences in measurement methods, and statistical analysis methods.

Therefore, our study aimed to develop a multiple linear regression model to predict HRPF parameters (e.g., HGS, flexibility, muscular endurance, and CRF) using easy-to-measure dependent variables [e.g., gender, age, body mass index (BMI), and percent body fat] in Korean adults.

## Materials and Methods

### Datasets

The National Fitness Award (NFA) datasets of South Korea were used in this analysis. The NFA is a nationwide test in 75 sites that assesses the physical fitness of the general population in South Korea. This study included male and female (age: 19–64 years) who participated in the NFA from 2015 to 2019. Among a total of 457,942 adults, we excluded participants who had no data on their dependent variables (*n* = 640) and had no data on their HRPF parameters (*n* = 669). Finally, a total of 456,633 adults (male: *n* = 210,613, female: *n* = 246,020) were included in the analysis. Male and female were divided in the ratio of 7:3 using the Bernoulli trial. Approximately 70% of the divided data (total: *n* = 319,643, male: *n* = 147,600, female: *n* = 172,043) were used in the development of the HRPF estimation formula with gender, age, BMI, and percent body fat, and approximately 30% of the data (total: *n* = 136,990, male: *n* = 63,013, female: *n* = 73,977) were used for the validity test. The power test was performed using G^∗^Power 3.1.9.2 (Franz Faul, University of Kiel, Kiel, Germany) at the tails of two, the H1 ρ^2^ of 0.3, the H0 ρ^2^ of 0, the significant level of 0.05 (α = 0.05), the power of 0.9, and the number of predictors of 4 for all statistical tests. G^∗^Power showed that 51 subjects had sufficient power for this study. The study was conducted according to the guidelines of the Declaration of Helsinki and approved by the Institutional Review Board of Kunkuk University (7001355-202101-E-132). All individuals provided informed consent before enrollment. The population characteristics are presented in Table 1.

**TABLE 1:** Characteristics of the study population.

Variables	Regression model data			Validity test data		
	Total (*n* = 319,643)	Male (*n* = 147,600)	Female (*n* = 172,043)	Total (*n* = 136,990)	Male (*n* = 63,013)	Female (*n* = 73,977)
Age (years)	38.95 ± 14.91	35.16 ± 14.21	42.20 ± 14.74	38.91 ± 14.89	35.07 ± 14.17	42.18 ± 14.72
Height (cm)	165.42 ± 9.09	172.79 ± 6.25	159.09 ± 5.78	165.40 ± 9.10	172.83 ± 6.24	159.07 ± 5.75
Bodyweight (kg)	65.24 ± 12.39	73.57 ± 10.90	58.10 ± 8.54	65.23 ± 12.38	73.55 ± 10.80	58.14 ± 8.67
BMI (kg/m^2^)	23.73 ± 3.35	24.61 ± 3.18	22.97 ± 3.30	23.73 ± 3.36	24.60 ± 3.16	23.00 ± 3.34
Percent body fat (%)	26.45 ± 8.15	21.23 ± 6.65	30.92 ± 6.48	26.48 ± 8.16	21.22 ± 6.64	30.96 ± 6.49
HGS (kg)	31.18 ± 10.39	40.17 ± 7.57	23.47 ± 4.73	31.14 ± 10.38	40.14 ± 7.59	23.47 ± 4.73
Sit and reach (cm)	12.00 ± 9.35	8.80 ± 9.42	14.75 ± 8.37	12.01 ± 9.33	8.83 ± 9.41	14.71 ± 8.35
Sit-ups (n)	30.57 ± 15.95	40.16 ± 13.73	22.33 ± 12.82	30.56 ± 15.94	40.24 ± 13.67	22.30 ± 12.78
Estimated VO_2*max*_ (ml/kg/min)	36.25 ± 6.74	40.91 ± 5.94	31.95 ± 4.04	36.25 ± 6.77	40.94 ± 5.94	31.91 ± 4.04

*Values are expressed as mean ± SD. BMI, body mass index; HGS, hand grip strength; VO_2max_, maximal oxygen uptake.*

### Measurement of Dependent Variables

Height was measured to the nearest 0.1 cm using a stadiometer (Seca, Seca Corporation, Columbia, MD, United States). Body weight and percent body fat were measured using bioelectrical impedance analysis equipment (Inbody 720, Inbody, Seoul, Korea) (Jeong et al., 2020). BMI was calculated by dividing body weight (kg) by height squared (m^2^).

### Health-Related Physical Fitness Parameters

All HRPF parameters were measured by certified health and physical fitness instructors. The HRPF assessment for adults included HGS, flexibility (sit and reach), muscular endurance (sit-ups), and CRF (estimated VO_2*max*_). Descriptions of the tests are as follows:

HGS (kg): Isometric muscle strength was assessed using a hand dynamometer (GRIP-D 5101, Takei, Niigata, Japan). Participants held the dynamometer with their preferred hand and squeezed it as forcefully as possible. All participants were tested twice, and the best result was recorded to the nearest 0.1 kg.

Sit-and-reach (cm): The participants sat on a mat and placed their feet in front of the measurement board with their legs fully extended. Participants were directed to gradually reach forward with both hands overlapped and push the bar as far as possible, holding this position for approximately 3 s. The best score was recorded after two trials and recorded to the nearest 0.1 cm.

Sit-ups (number of times): The participants laid on a mat with their knees bent at 90° and their feet held down by a partner. After being instructed to begin, they raised their upper body until their elbows touched the knees, and then returned to the initial position where both shoulders were in contact with the mat. Their hands were required to remain placed crosswise on the chest during the test. The total number of accurately performed and complete sit-ups was recorded.

Estimated VO_2*max*_ (ml/kg/min): A graded exercise treadmill test with Bruce protocol (Bruce et al., 1973) was applied to measure a VO_2*max*_. All participants began walking at a speed of 2.7 km/h, at an inclination of 10%. The speed was increased 1.3–1.4 km/h at 3 min intervals, and the incline was increased by 2% with each stage. The graded exercise test was performed on a treadmill (TM55 treadmill, Quinton Cardiology Systems, Inc., Seattle, WA, United States). Heart rate was measured using a heart rate monitor (Quinton Q-Stress, Quinton Cardiology Systems, Inc., Bothell, WA, United States). The participants were expected to reach three of the following criteria: (1) heart rate reserve >85%; (2) heart rate did not increase even when the stage increased; (3) rating of perceived exertion >17 (range: 6–20); (4) request to stop by the participant. The VO_2*max*_ was calculated using the Bruce formula: 6.70 − 2.82 × (1: male, 2: female) + (0.056 × exercise maintaining time (s)) (Bruce et al., 1973).

### Statistical Analysis

The mean and standard deviation were calculated for all measured parameters. The normality of distribution of all outcome variables was verified using the Kolmogorov–Smirnov test. To perform multiple linear regression analysis, the β-value (the regression coefficient) was used to verify if the independent variables had explanatory power (Park et al., 2020). In this work we used the stepwise mode of regression analysis, which is indicated when multiple independent variables are taken as predictors (Shepperd and MacDonell, 2012; Bardsiri et al., 2014). The stepwise regression technique aims to maximize the estimated power with a minimum number of independent variables. Multiple linear regression analysis with the stepwise technique predicted HRPF parameters (HGS, flexibility, muscular endurance, and CRF) using dependent variables (e.g., gender, age, body mass index, and percent body fat). In addition, we rigorously conformed to the basic assumptions of the regression model: linearity, independence, autocorrelation, homoscedasticity, continuity, normality, and outliers. The outlier data in the multiple regression model were identified and removed when the absolute value of the studentized residual (SRE) was ≥2. The validity of the regression model was tested using approximately 30% of the total data, which had already been divided through the Bernoulli trial, and were not included in the development of the regression model. The validation test calculated the predicted values of the HRPF parameters using the regression equation, and the mean error and standard errors of estimation (SEE) were calculated using formulas 1 and 2. Two-tailed Pearson-correlation analysis was performed to estimate the relationships between measured and predicted HRPF parameters. The Statistical Package for the Social Sciences (SPSS) version 25.0 (IBM Corporation, Armonk, NY, United States) was used for analysis, and the level of significance was set at 0.05.

$$Meanerror\left(\%\right)=\frac{\sum \frac{Measuredvalue-Predictedvalue}{Measuredvalue}*100}{N}$$

**Formula 1.** The calculation formula for the mean error

$$Standarderrorsofestimation=\frac{\sum {\left(Mesuredvalue-Predictedvalue\right)}^{2}}{N-2}$$

**Formula 2.** The calculation formula for the standard errors of estimation.

## Results

For each multiple regression model developed, the F-test was used to validate the significance of the model. Multiple regression analyses have shown that the regression coefficients for the selected independent variable were statistically significant. Multiple regression analyses for each model included coefficients of determination (*R*^2^), adjusted coefficients of determination (adjusted *R*^2^), and SEE. The correlations between the dependent variables and HRPF parameters are shown in Table 2.

**TABLE 2:** Correlation coefficients between dependent variables and HRPF parameters for the estimating regression model.

Dependent variables		HRPF parameters			
		HGS	Sit and reach	Sit-ups	Estimated VO_2*max*_
Gender	R	−0.802*	0.316**	−0.558**	−0.665**
Age	R	−0.262**	0.058**	−0.523**	−0.545**
BMI	R	0.278**	−0.154**	−0.057**	−0.207**
Percent body fat	R	−0.558**	0.021**	−0.626**	−0.749**

Significant correlation between measured HRPF parameters and dependent variables. HRPF: health-related physical fitness; BMI: body mass index; HGS: hand grip strength; VO_2max_: maximal oxygen uptake. **P < 0.01.

### Performance Evaluation of Regression Models and Regression Equations

The detailed results of the multiple regression analysis using HRPF parameters are shown in Table 3. The estimated explanatory power of HGS regression models was 71.0%, and SEE was 5.60 kg (*F* = 194,597.062, *P* < 0.001). Further, the explanatory power of the sit and reach regression models was 15.5%, and SEE was 8.60 cm (*F* = 14,568.080, *P* < 0.001). The explanatory power of sit-ups regression models was 55.5%, and SEE was 10.63 n (*F* = 98,806.560, *P* < 0.001). In addition, the explanatory power of estimated VO_2*max*_ regression models was 72.0%, and SEE was 3.56 ml/kg/min (*F* = 131291.452, *P* < 0.001).

**TABLE 3:** Estimated regression equations predicting HRPF parameters.

Regression model	*R*	*R*^2^	Adjusted *R*^2^	*F-*value	*P* value	SEE
HGS = 35.264 − (9.668 × gender) − (0.513 × percent body fat) + (1.064 × BMI) − (0.044 × age)	0.842	0.710	0.710	194,597.062	0.000	5.60
Sit and reach = −3.071 + (10.812 × gender) − (0.451 × percent body fat) + (0.397 × BMI) + (0.024 × age)	0.393	0.155	0.155	14,568.080	0.000	8.60
Sit-ups = 59.556 − (0.933 × percent body fat) − (0.367 × age) + (0.742 × BMI) − (4.983 × gender)	0.745	0.555	0.555	98,806.560	0.000	10.63
Estimated VO_2*max*_ = 62.782 − (0.279 × percent body fat) − (0.135 × age) − (5.555 × gender) − (0.242 × BMI)	0.848	0.720	0.720	131,291.452	0.000	3.56

*HRPF, health-related physical fitness; SEE, standard error of estimation; gender: 1 = male, 2 = female; BMI, body mass index; HGS, hand grip strength; VO_2max_, maximal oxygen uptake.*

### Performance Evaluation of Regression Models and Regression Equations Without Outlier Data

Table 4 shows the results of the multiple regression analysis using HRPF parameters without outlier data. The explanatory power of HGS regression models (SRE 27: n = 253,339) was 87.0%, and SEE was 3.27 kg (*F* = 422009.836, *P* < 0.001). Moreover, the explanatory power of the developed sit and reach regression models (SRE 31: *n* = 263,737) was 29.8%, and SEE was 5.64 cm (*F* = 28,019.748, *P* < 0.001). The explanatory power of sit-ups regression models (SRE 34: *n* = 268,182) was 75.1%, and SEE was 7.44 n (*F* = 202,721.241, *P* < 0.001). In addition, the explanatory power of estimated VO_2*max*_ regression models (SRE 44: *n* = 151,314) was 88.5%, and SEE was 1.77 ml/kg/min (*F* = 290,332.119, *P* < 0.001).

**TABLE 4:** Estimated regression equations predicting HRPF parameters without outlier data.

Regression model	R	R^2^	Adjusted R^2^	*F-*value	*P* value	SEE
HGS (SRE 27: n = 253,339) = 37.138 − (10.190 × gender) + (0.988 × BMI) − (0.457 × percent body fat) − (0.042 × age)	0.932	0.870	0.870	422,009.836	0.000	3.27
Sit and reach (SRE 31: n = 263,737) = 0.005 + (10.762 × gender) − (0.432 × percent body fat) + (0.339 × BMI) + (0.009 × age)	0.546	0.298	0.298	28,019.748	0.000	5.64
Sit-ups (SRE 34: n = 268,182) = 62.443 − (1.015 × percent body fat) − (0.392 × age) + (0.783 × BMI) − (5.287 × gender)	0.867	0.751	0.751	202,721.241	0.000	7.44
Estimated VO_2*max*_ (SRE 44: n = 151,314) = 61.068 − (0.197 × percent body fat) − (5.920 × gender) − (0.133 × age) − (0.305 × BMI)	0.941	0.885	0.885	290,332.119	0.000	1.77

*HRPF, health-related physical fitness; SEE, standard error of estimation; SRE, studentized residual; gender: 1 = male, 2 = female; BMI, body mass index; HGS, hand grip strength; VO_2max_, maximal oxygen uptake.*

### Regression Model Validity

The validity of the developed regression models was calculated using data not included in multiple regression analyses. In all regression models of HRPF parameters, the mean error was −38.13 to 3.36% (HGS: −4.33%, sit and reach: −14.92%, sit-ups: −38.13%, and estimated VO_2*max*_: 3.36%), and SEE was higher than the developed regression model (Table 5).

**TABLE 5:** Validity of estimating the regression model.

	HGS	Sit and reach	Sit-ups	Estimated VO_2*max*_
Mean error (%)	−4.33	−14.92	−38.13	3.36
SEE	5.61 kg	8.72 cm	10.65 n	3.92 ml/kg/min

*SEE, standard error of estimation; HGS, hand grip strength; VO_2max_, maximal oxygen uptake.*

### Relationship Between Measured and Predicted HRPF Parameters

**FIGURE 1:**
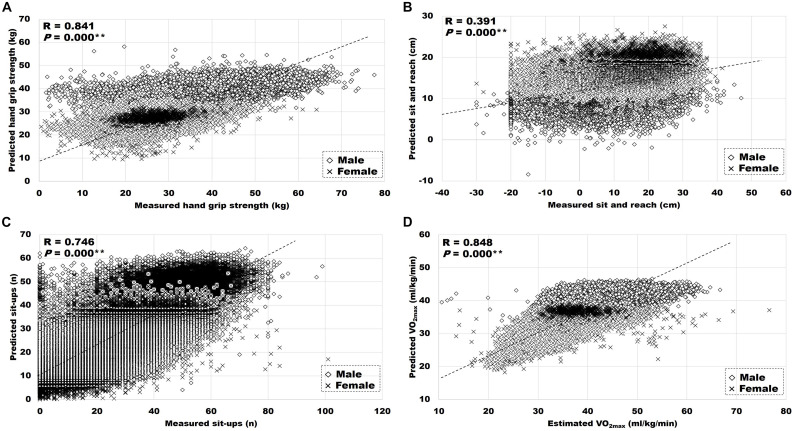
Relationship between measured or estimated, and predicted HRPF. **(A)** Hand grip strength. **(B)** Sit and reach. **(C)** Sit-ups. **(D)** VO_2*max*_. Significant correlation between measured or estimated and predicted variables, **P < 0.01.

Table 6 displays the relationship between the measured and predicted HRPF parameters. Measured HRPF parameters were positively related with predicted HGS (*r* = 0.841, *P* < 0.01), sit and reach (*r* = 0.391, *P* < 0.01), sit-ups (*r* = 0.746, *P* < 0.01), and estimated VO_2*max*_ (*r* = 0.848, *P* < 0.01), as seen in Figure 1.

**TABLE 6:** Relationship between measured and predicted HRPF parameters.

HRPF parameters	*R*	*P* value
HGS	0.841	0.000**
Sit and reach	0.391	0.000**
Sit-ups	0.746	0.000**
Estimated VO_2*max*_	0.848	0.000**

Significant correlation between measured and predicted HRPF parameters, **P < 0.01. HRPF, health-related physical fitness; HGS, hand grip strength; VO_2max_, maximal oxygen uptake.

## Discussion

Over the years, the components of HRPF have been established in various ways in scientific research (Meredith and Welk, 2010). Previous studies describe HRPF as having a multidimensional structure despite the many different definitions (Meredith and Welk, 2010). Some European studies consider HRPF to include body composition, musculoskeletal fitness, CRF, and skill-related fitness (agility, speed, and coordination) (Artero et al., 2011; Ruiz et al., 2011; Secchi et al., 2014). Other studies consider only body composition, CRF, musculoskeletal fitness, and flexibility (Pillsbury et al., 2013); or body composition, CRF, muscle strength, and flexibility as components of HRPF (Castillo-Garzón et al., 2006). However, the American College of Sports Medicine recommends five factors: body composition, flexibility, muscular strength, muscular endurance, and CRF (Liguori and American College of Sports Medicine, 2020). Therefore, multiple regression analysis using the stepwise technique predicted the HRPF parameters (HGS, flexibility, muscular endurance, and CRF) of the American College of Sports Medicine criteria using dependent variables (e.g., gender, age, body mass index, and percent body fat).

Many researchers have conducted studies to evaluate health conditions and exercise performance using HRPF, while assuming that the HRPF parameter is a reliable healthcare index. For healthcare, the development of tools or equipment that can easily measure and evaluate HRPF in daily life will be useful. Previous studies developed equations with relatively small sample sizes or samples with limited age ranges (Esco et al., 2008; Shenoy et al., 2012; Lopes et al., 2018; Zaccagni et al., 2020). This study aimed to develop a multiple regression model for estimating the HRPF parameters in Korean adults using easy-to-measure dependent variables. Before performing multiple regressions to estimate HRPF parameters, it is essential to eliminate outliers because they increase predictive errors. The absolute value of the studentized residual was used to eliminate outliers in this study. The coefficient of determination of the HRPF parameters in the developed multiple regression models was high, except for flexibility. The mean explanatory power of the sit and reach regression model in our study was 29.8%.

The HGS used to evaluate total muscle strength measures the ability of hand muscles to produce force (tension) using a hand dynamometer (Mitsionis et al., 2009). The relevance of HGS measurements continues to grow due to their clinical and epidemiological application for sarcopenia diagnosis, as suggested by the European Working Group (Cruz-Jentoft et al., 2010), or as a nutrition status indication and their association with morbidity and mortality (Norman et al., 2011). HGS has been studied in relation with various anthropometric factors (Alahmari et al., 2017; Eidson et al., 2017; Lopes et al., 2018; Zaccagni et al., 2020). In the current study, the mean explanatory power of the HGS regression model (37.138 − (10.190 × gender_*male* = 1; *female* = 2_) + (0.988 × BMI) − (0.457 × percent body fat) − (0.042 × age)) was 87.0% (adjusted *R*^2^). Alahmari et al. (2017) showed that three variables (i.e., age, hand length, and forearm circumference) predicted 42.7% (adjusted *R*^2^) of what constitutes the HGS of healthy adult males (aged: 20–74 years; *n* = 116) in Saudi Arabia. Furthermore, Zaccagni et al. (2020) reported that the independent variables sex, upper arm muscle area, arm fat index, fat mass, and fat free mass accounted for 74.6% (adjusted *R*^2^) of the variance of HGS in young adults (aged: 18–30 years; total: *n* = 544; male: *n* = 356; female: *n* = 188). Lopes et al. (2018) showed that 71% (adjusted *R*^2^) of the variability in the dominant HGS could be explained by gender, forearm circumference, and hand length (−15.490 + (10.787 × gender_*male* = 1; *female* = 0_) + (0.558 × forearm circumference) + (1.763 × hand length)). In addition, 70% (adjusted *R*^2^) of the variability in the nondominant HGS was explained by gender and hand length (−9.887 + (12.832 × gender_*male* = 1; *female* = 0_) + (2.028 × hand length)) in young adult and middle-aged participants (aged: 20–60 years; total: *n* = 80; male: *n* = 40; female: *n* = 40). Our study confirmed that the regression model formulation developed is more accurate and straightforward than the predictive power of previous studies.

The two most important trunk muscle abilities have been presented as trunk muscle strength and muscular endurance in both the athletic and general populations (Granacher et al., 2013). Trunk muscle strength and muscular endurance testing in clinical fields have been important in injury rehabilitation and prevention programs (Jackson et al., 1998; del Pozo-Cruz et al., 2013). Sit-ups test are known to evaluate strength and muscular endurance in the abdomen (Morrow et al., 2015; Liguori and American College of Sports Medicine, 2020). Esco et al. showed that 63.7% (*R*^2^) of the variability in sit-ups could be explained by height, push-ups, skinfolds at the thigh, and skinfolds at the subscapularis (1.651 + (0.368 × push-ups) + (0.495 × height) − (0.277 × skinfolds at the thigh) − (0.336 × skinfolds at the subscapularis)) in healthy adults (aged: 18–48 years; total: *n* = 100; male: *n* = 40; female: *n* = 60) (Esco et al., 2008). The sit-ups regression model’s (62.443 − (1.015 × percent body fat) − (0.392 × age) + (0.783 × BMI) − (5.287 × gender_*male* = 1; *female* = 2_)) mean explanatory power estimated in our study was 75.1% (adjusted *R*^2^).

Cardiorespiratory fitness is an essential component of health and physical fitness, and is affected by the respiratory, cardiovascular, and skeletal muscle systems (Liguori and American College of Sports Medicine, 2020). The gold standard measurement of CRF is VO_2*max*_ when performing a maximum graded exercise test (Liguori and American College of Sports Medicine, 2020). However, while VO_2*max*_ is the most accurate way to evaluate CRF, testing requires expensive equipment, space to accommodate equipment, and trained personnel. Previous studies developed a method to predict VO_2*max*_ without exercise using multiple regression analysis (Bradshaw et al., 2005; Shenoy et al., 2012). The non-exercise regression equations provide convenient estimates of CRFs without performing maximum or submaximal exercise tests (Bradshaw et al., 2005). Shenoy et al. showed that 79.9% (adjusted *R*^2^) of the variability in VO_2*max*_ could be explained by gender, perceived functional ability, and body surface area (−1.541 + (1.096 × gender_*male* = 1; *female* = 0_) + (0.081 × perceived functional ability) + (1.084 × body surface area)) in healthy young Indian adults (aged: 18–27 years; total: *n* = 120; male: *n* = 60; female: *n* = 60) (Shenoy et al., 2012). Bradshaw et al. (2005) showed that 87% (*R*^2^) of the variability in VO_2*max*_ could be explained by gender, age, BMI, perceived functional ability, and PA rating (48.073 + (6.178 × gender_*male* = 1; *female* = 0_) – (0.246 × age) – (0.619 × BMI) + (0.712 × perceived functional ability) + (0.671 × PA rating)) in adults (aged: 18–65 years; total: *n* = 100; male: *n* = 50; female: *n* = 50). In our study, the mean explanatory power of the estimated VO_2*max*_ regression model (61.068 − (0.197 × percent body fat) − (5.920 × gender_*male* = 1; *female* = 2_) − (0.133 × age) − (0.305 × BMI)) was 88.5% (adjusted *R*^2^). Accordingly, we obtained similar or higher regression coefficient than previous studies by using independent variables that are more accessible to measure, and a larger sample size. Therefore, we consider the results of this study straightforward and accurate.

## Limitations

This study had some limitations. The role of HRPF and nutrition in decreasing the progression of chronic diseases is growing more important (Gil et al., 2015). Nutrition was described as a major modifiable behavior, and HRPF has also been defined as an essential health-related indication (Camões and Lopes, 2008). Previous studies have shown that improvements in HRPF and nutritional factors could prevent functional limitations related to aging, lead to healthier and independent aging processes (Strandberg et al., 2017; Wickramasinghe et al., 2020). However, the association with HRPF parameters could not be evaluated because the NFA database did not provide nutrition information. We only included adults between the ages of 19 and 64 in our analysis. Therefore, the multiple regression equation developed in the present study does not apply to older adults. In the future, a multi-regression equation development study will be necessary to predict the functional physical fitness of older adults.

## Conclusion

This study demonstrated that the variability of HGS, muscular endurance, and CRF in healthy adults could be explained by gender, age, BMI, and percent body fat. A multi-regression equation could be developed based on these demographic and anthropometric variables. Since this multi-regression equation requires only a simple parameter measurement, it could be time-efficient, inexpensive, and realistic for large groups in clinical practice. The prediction equation will allow coaches, athletes, healthcare professionals, researchers, and the general public to better estimate the expected HRPF in order to improve the data interpretation.

## Data Availability Statement

The original contributions presented in the study are included in the article/Supplementary Material, further inquiries can be directed to the corresponding author.

## Ethics Statement

The studies involving human participants were reviewed and approved by Institutional Review Board of Kunkuk University. Written informed consent for participation was not required for this study in accordance with the national legislation and the institutional requirements.

## Author Contributions

S-WK, HJ, JL, and H-YP: conception and study design. S-WK, HJ, and H-YP: statistical analysis. H-YP: investigation. S-WK and H-YP: data interpretation and writing–review and editing. S-WK: writing–original draft preparation. KL: supervision. All authors have read and approved the final manuscript.

## Conflict of Interest

The authors declare that the research was conducted in the absence of any commercial or financial relationships that could be construed as a potential conflict of interest.
